# From Nucleobases to Bioactive NHC‐Based Gold and Silver Complexes With Anticancer and Antiviral Activity

**DOI:** 10.1002/cmdc.70447

**Published:** 2026-08-25

**Authors:** Domenico Iacopetta, Jessica Ceramella, Paola Checconi, Flavio Frezza, Maria Marra, Annaluisa Mariconda, Assunta D’Amato, Alessia Catalano, Stefano Aquaro, Pasquale Longo, Maria Stefania Sinicropi

**Affiliations:** ^1^ Department of Pharmacy Health and Nutritional Sciences University of Calabria Arcavacata di Rende Italy; ^2^ Department for the Promotion of Human Sciences and Quality of Life San Raffaele University Rome Italy; ^3^ Laboratory of Microbiology IRCCS San Raffaele Roma Rome Italy; ^4^ Department of Basic and Applied Science University of Basilicata Potenza Italy; ^5^ Department of Chemistry and Biology University of Salerno Fisciano Italy; ^6^ Department of Pharmacy‐Drug Sciences University of Bari “Aldo Moro” Bari Italy; ^7^ Department of Life Health and Environmental Sciences University of L’Aquila L’Aquila Italy

**Keywords:** anticancer activity, antiviral activity, *N*‐heterocyclic carbene (NHC) complexes, nucleobases

## Abstract

*N*‐heterocyclic carbene (NHC) ligands are known for their exceptional versatility and applications, being good σ‐donors, sterically tunable and quite stables at different thermic, pH and oxidative conditions. Furthermore, they have been proved to be excellent ligands for transition metals and, particularly, gold (Au) and silver (Ag) complexes have been intensively studied for their biomedical and therapeutic applications. Both NHC‐Au‐ and ‐Ag complexes have been shown to target selectively cancer cells or bacteria, without exerting side or unwanted effects on the normal cells’ viability. Herein we reported the design, synthesis and biological evaluation of a new series of NHC‐Au and ‐Ag complexes, adopting nucleobases, that is guanine and guanosine, as NHC precursors. The individuated lead, which contains gold, was able to hamper, particularly, the growth of MDA‐MB‐231 breast cancer cells, inducing genomic DNA damage and apoptosis. Additionally, the same complex exerted an antiviral activity in bronchial epithelial cells infected with influenza virus A/H1N1. The disclosed double biological activity offers new hints in the field of synthesis and pharmaceutical use of biohybrid organometallic complexes, as valid scaffolds for the potential treatment of oncologic patients with viral infection complications or viceversa to hamper the tumor onset after viral infections.

## Graphical abstract

Herein, we reported the design and synthesis of a novel series of *N*‐heterocyclic carbene (NHC) complexes originated from the imidazole ring of guanine and guanosine and with different counterions (iodide, acetate, and aminoacidate). The evaluation of the biological activities, that is anticancer and antiviral, indicated **L2AuOAc** as the best complex, which bears a gold(I) center, a ribose moiety and an acetate counterion.

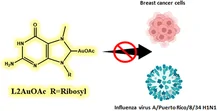

## 1 Introduction

In recent years, metal complexes stabilized by *N*‐heterocyclic carbenes (NHCs) have demonstrated remarkable potential in both catalytic applications and, more recently, in the field of medicinal chemistry [1, 2]. Particularly, they have assumed an increasing important role in organometallic and pharmaceutical chemistry because of their tunable stability and electronic properties, being also excellent ligands for transition metals [3, 4]. NHCs can be synthetically obtained from different starting materials, for instance caffeine and theobromine, and can strongly bind several transition metals, obtaining complexes with antibacterial, anticancer, antioxidant and anti‐inflammatory properties [5, 6, 7, 8, 9, 10, 11]. Several targets have been found to be modulated by NHC‐metal complexes as, to name but a few, thioredoxin reductase, human topoisomerases I and II, actin, tubulin, and so on [12, 13, 14]. Over the years, our research group has focused the attention on the design, synthesis, characterization, and biological evaluation of numerous silver, gold, and ruthenium complexes stabilized by NHC ligands [11, 15, 16, 17, 18, 19, 20, 21]. We have systematically varied their lipophilic and hydrophilic properties to establish a correlation between molecular structure and biological activity. Our ongoing research continues in this direction, aiming to introduce novel structural modifications to the carbene ligands. For instance, by tuning precisely the electronic and steric properties of the NHC scaffold, it is possible to enhance the stability of the metal–ligand bond and optimize the cellular uptake of the complexes. These strategic modifications are directed toward the improvement of the pharmacological profile, potentially increasing the biological efficacy while minimizing off‐target toxicity. In this context, the use of nucleobases as NHC precursors represents a particularly interesting and innovative idea and amongst them, guanine and guanosine emerge as promising scaffolds. Indeed, they feature a bicyclic system containing an intrinsic imidazole ring, which constitutes a natural precursor for the generation of stable NHCs, while maintaining useful functionalities for molecular recognition and interaction with biological systems [22, 23, 24]. Several studies have demonstrated that modifications of the purine ring can lead to the formation of imidazolium salts derived from nucleobases, direct precursors of NHCs, paving the way for the synthesis of biohybrid organometallic complexes [25, 26]. The integration of such ligands with noble metals allows to combine the chemical properties of carbenes with the biocompatibility and potential selectivity of nucleobases. In particular, gold(I) and silver(I) complexes containing nucleoside‐derived NHCs have shown interesting biological properties, including anticancer and antimicrobial activities, often attributed to their ability to interact with biomolecules and enzymatic systems [27, 28, 29]. In this work, we explored synthetic methodologies for the generation of NHCs originating from the imidazole rings of guanine and guanosine, and their subsequent coordination to noble metals, such as gold(I) and silver(I). The resulting complexes were investigated to evaluate their structural properties and pharmacological potential, with the aim of contributing to the development of new organometallic systems that exploit natural scaffolds for innovative therapeutic applications. It is worth noting that the coordination of the NHC to the metal center occurs through the C8 carbon of the purine scaffold; this preserves the sites involved in the Watson–Crick base pairing, allowing for selective interactions with biomolecules. Literature data and some of our previously published results indicated a high potential of NHC‐silver and ‐gold complexes in diminishing the proliferation of different cancer cells and, as well, as antimicrobials. Thus, the new series of guanosine‐ and guanine‐derived *N*‐heterocyclic carbene silver(I) and gold(I) complexes were mainly screened for their anticancer and antiviral properties. The most active complex was shown able to decrease the viability of two breast cancer (MCF‐7 and MDA‐MB‐231) and one epithelial cervix carcinoma (HeLa) cell lines, without any effect on the viability of the normal breast MCF‐10A cells. The exposure of this complex to MDA‐MB‐231 cells induce genomic DNA fragmentation and, consequently, cancer cells death by the intrinsic apoptotic mechanism. Moreover, its anti‐influenza activity in bronchial epithelial cells infected with influenza virus A/Puerto Rico/8/34 H1N1 was also determined. It is known that cancer patients may be exposed to a high risk for respiratory and, in particular, influenza viruses’ complications [30, 31], because of the weakened immune system. Furthermore, a chronic exposure to the influenza virus and/or a severe pneumonia virus infection may increase the risk of cancer [31, 32]. Thus, our outcomes can provide interesting insights for the development of scaffolds with a double activity, which will need further investigations in vivo, for facing both cancer and viral infections.

## 2 Results and Discussion

The medicinal deployment of metal complexes incorporating guanine and guanosine ligands exploits biomimetic strategies to enhance DNA‐binding affinity and circumvent systemic toxicity associated with classical metallodrugs. Recent studies have demonstrated that ruthenium(II) and palladium(II) conjugates, particularly those featuring guanosine‐substituted architectures, demonstrate superior cytotoxicity against resistant neoplastic cell lines through G‐quadruplex stabilization. The use of nucleobase derivatives as therapeutic agents further suggests that their incorporation in a metal complex may provide a wide array of pharmaceutical applications, particularly when bound to the metal center as NHC. Furthermore, the strategic functionalization of guanosine, such as the introduction of 2^′^,3^′^,5^′^‐tri‐O‐acetyl protecting groups, has been shown to significantly improve the lipophilicity and cellular uptake of platinum(II) and palladium(II) complexes [26, 33]. The diverse coordination modes and geometries afforded by these purine‐based ligands allow for the precise modulation of the metal centre’s reactivity [34]. Such biochemical versatility highlights these guanine‐based hybrids as promising scaffolds for the development of high‐selectivity chemotherapeutics with reduced off‐target effects. Consequently, the integration of nucleoside ligands into organometallic frameworks represents a pivotal advancement in the design of next‐generation, bio‐inspired oncological interventions.

### 2.1 Synthesis of Silver(I) and Gold(I) Complexes

**SCHEME 1:**
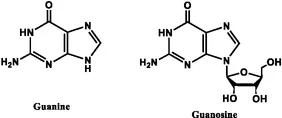
Guanine and guanosine used as NHC precursors in this work.

The syntheses of the new metal complexes were performed by introducing structural modifications to the selected synthons. Specifically, guanine and guanosine (Scheme 1) were subjected to alkylation with iodomethane to afford the corresponding imidazolium salts. These intermediates serve as suitable precursors for the stabilization of metal ions through the subsequent in situ generation of NHC ligands.

### 2.2 Synthesis of Silver(I) Complexes

**SCHEME 2:**
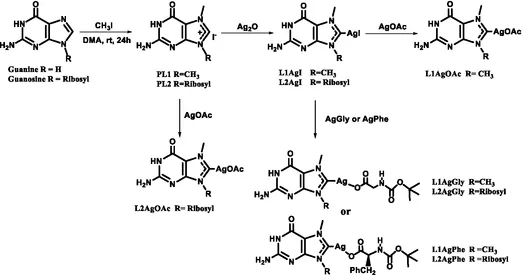
Synthesis of silver(I) complexes.

Silver complexes were synthesized as reported in Scheme 2.

The synthesis of the imidazolium salts **PL1** and **PL2** was carried out under an inert nitrogen atmosphere, by reacting guanine or guanosine with an excess of iodomethane in *N*,*N*’‐dimethylacetamide at room temperature for 24 h. Under these conditions, the reaction proceeds through the mono‐ or dialkylation of the nitrogen atoms present in the purine scaffold, leading to the formation of the corresponding imidazolium salts. The use of an excess of the alkylating agent promotes efficient quaternization of the nucleobase precursor and facilitates the formation of stable NHC precursors. Following completion of the reaction, the crude products were isolated by the addition of methanol to the reaction mixture, which was subsequently treated with diethyl ether to induce precipitation of the desired salts. The resulting solids were collected and purified according to a procedure adapted from the literature [35].

The ^1^H‐NMR characterization of proligand **PL1** exhibits signals corresponding to the methyl protons at chemical shift values of 3.6_9_ ppm and 3.9_9_ ppm; a further characteristic signal is that of the proton bonded to the cationic (C2) carbon, resonating at 9.1_4_ ppm. In the ^13^C‐NMR spectrum, two signals are observed at 31.2_5_ ppm and 35.2_4_ ppm relating to the methyl carbons, while the carbocationic carbon resonates at 138.5_3_ ppm. As for **PL2**, the ^1^H‐NMR spectrum shows a methyl signal at 4.0_1_ ppm, while the proton bonded to the cationic carbon resonates at 9.3_4_ ppm. In the ^13^C‐NMR spectrum, the methyl signal is observed at 35.7_2_ ppm, with the cationic carbon appearing at 136.3_7_ ppm.

**FIGURE 1:**
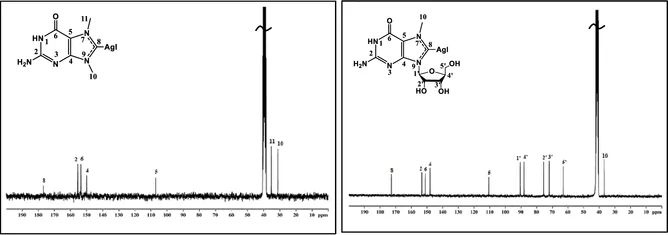
^13^C‐NMR spectra of L1AgI and L2AgI.

Iodide silver(I) complexes **L1AgI** and **L2AgI** were obtained by treating the corresponding proligand with silver oxide, as reported in the literature [36]. Successful metalation was attested by spectroscopic analyses. The ^1^H‐NMR analysis shows the disappearance of the signal at 9.1_4_ ppm, corresponding to the proton bound to the cationic carbon, which demonstrates the deprotonation of the proligand. Successful complexation is further confirmed by ^13^C‐NMR spectroscopy. Comparing the ^13^C‐NMR spectra of the proligand (**PL1**) and the resulting complex reveals a significant downfield shift of the signal from 138.5_3_ ppm (carbocationic) to 177.8_3_ ppm, characteristic of a metal‐coordinated carbenic carbon (Figure 1).

Mass spectrometry analysis provided additional evidence, showing a base peak at *m/z* 467.07141, attributable to a bis‐carbene structure with two NHC ligands coordinated to a silver cation.

Similarly, proton spectrum of silver(I) salt **L2AgI** shows the disappearance of the procarbenic signal at 9.3_4_ ppm, while ^13^C‐NMR shows the marked downfield shift of the carbenic carbon, now appearing at 173.5_6_ ppm. Mass spectrometry analysis indicated, again, a bis‐carbenic structure with two NHC ligands coordinated to a single silver atom. These data collectively confirm the formation of the silver(I) bis‐carbene complexes **(NHC)_2_AgI** for both ligands.

Acetate silver(I) complexes **L1AgOAc** and **L2AgOAc** were synthesized according to the literature, by anionic exchange in the presence of silver(I) acetate. In the case of **L1AgOAc**, diagnostic ^13^C‐NMR shifts involved the presence of the methyl acetate carbon at 22.2_4_ ppm, along with an upfield shift to 174.8_5_ ppm of the carbenic carbon. The same accounts for complex **L2AgOAc**, which signals were observed at 23.8_5_ ppm and 169.8_6_ ppm, respectively.

Derivatives comprising an aminoacidic residue, namely glycine or L‐phenylalanine, were obtained using the same synthetic strategy as for complexes **L1/2AgOAc**. Complexes **L1AgI** or **L2AgI** were subjected to anionic exchange in the presence of *N*‐Boc‐protected silver‐aminoacidic derivatives. The desired complexes were formed according to procedures from the literature, with precipitation of silver iodide [21, 37]. Confirmation of successful anionic exchange was ensured by ^13^C‐NMR spectra, which showed marked upfield shifts of the carbenic carbon in all four cases (to 171.4_6_, 172.8_3_, 170.5_4_, and 171.0_7_ ppm, respectively). It is worth noting that the MALDI‐MS data indicate the presence of a [(NHC)_2_M]^+^ species, whereas elemental analyses reveal a 1:1:1 ligand–metal–counterion stoichiometry. These findings suggest that all complexes adopt an ionic formulation of the type [(NHC)_2_M]^+^[MX_2_]^−^ in the solid state. Based on these results, it can reasonably be proposed that, in solution, an equilibrium exists between the ionic species [(NHC)_2_M]^+^[MX_2_]^−^ and the corresponding neutral complex M(NHC)X.

### 2.3 Synthesis of Gold(I) Complexes

**SCHEME 3:**
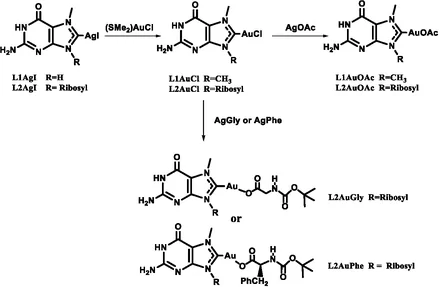
Synthesis of gold(I) complexes.

Gold(I) complexes were synthesized as reported in Scheme 3.

The gold complexes were obtained via transmetalation, starting from the silver complexes, **L1AgI** and **L2AgI**, which were treated with chloro(dimethyl sulfide)gold(I), (Me)_2_SAuCl, for 24 h in dry methanol.

**FIGURE 2:**
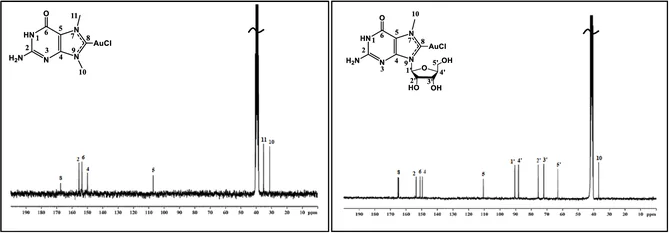
^13^C‐NMR spectra of L1AuCl and L2AuCl.

After 24 h, the product was recovered by first removing the solvent in vacuo, followed by washing with dichloromethane and methanol. The **L1AuCl** complex was characterized by ^1^H‐NMR, ^13^C‐NMR, and mass spectrometry. Specifically, the ^13^C‐NMR spectrum confirmed successful transmetalation due to the shift of the carbenic signal, which moves from 177.8_3_ ppm (corresponding to C‐Ag) to 167.8_2_ ppm (for C‐Au), in agreement with other complexes reported in the literature (Figure 2). Mass spectrometry also confirmed the structure for the gold complex, showing the gold(I) atom coordinated to two NHC ligands.

Similarly, for complex **L2AuCl**, the ^13^C‐NMR spectrum showed a marked upfield shift of the carbenic carbon from 173.5_6_ ppm to 164.5_1_ ppm, confirming the formation of the gold(I) bis–carbene complex (Figure 2).

Gold(I) acetate complexes **L1AuOAc** and **L2AuOAc** were obtained following the same strategy described for the silver(I) acetate ones. Anionic exchange, once again, was accomplished in the presence of silver(I) acetate, in refluxing dry methanol, overnight. Lastly, gold complexes with amino acid derivatives, such as glycinate (**L2AuGly**) and phenylalaninate (**L2AuPhe**), were obtained *via* counterion exchange from the corresponding gold‐chloride analogs. The reactions were carried out at room temperature in a methanol/acetonitrile solvent mixture. The target products were purified by filtration over Celite and isolated by removing the solvents under reduced pressure. Lastly, gold complexes with amino acid derivatives, such as glycinate (**L2AuGly**) and phenylalaninate (**L2AuPhe**), were obtained via counterion exchange from the corresponding gold‐chloride analogs. The reactions were carried out at room temperature in a methanol/acetonitrile solvent mixture. The target products were purified by filtration over Celite and isolated by removing the solvents under reduced pressure. The formation and purification of both products were confirmed by ^1^H‐NMR spectra, with the assignment of new signals corresponding to the amino acid derivatives. Specifically, the methylene protons resonate at 3.5_4_–3.4_2_ ppm for **L2AuGly** and 3.0_4_–2.8_8_ ppm for **L2AuPhe**. Additionally, the ^13^C‐NMR spectra confirmed the complex structures, with the methylene carbons appearing at 43.7_6_ ppm for **L2AuGly** and 37.5_2_ ppm for **L2AuPhe**.

### 2.4 Anticancer Activity

The anticancer potential of the new series of guanosine‐ and guanine‐derived NHC‐silver(I) and ‐gold(I) complexes was evaluated in breast and uterine cancer cells, namely the estrogen receptor α (ERα)‐positive MCF‐7 cells, the triple negative (ERα‐negative, PR‐negative and HER‐2/Neu not amplified) MDA‐MB‐231 cells and the epithelial cervix carcinoma Hela cells (ERα‐negative), by the means MTT assay, after 72 h of exposure. The calculated IC_50_ values, resumed in Table 1, indicate that the best activity was recorded for **L2AuOAc** in MDA‐MB231 cells, with an IC_50_ value of 17.0 ± 0.3 µM, a fair activity against the HeLa cells (49.0 ± 1.1 µM), whereas a lower effect in MCF‐7 cells (142.9 ± 2.3 µM) was observed. Furthermore, it did not hamper the viability of the normal breast MCF‐10A cells resulting, thus, selective for the considered cancer cells. More notably, its analogous silver complex **L2AgOAc** did not produce any effect toward all the cell lines used in this assay (IC_50_ > 200 µM), suggesting that the gold is essential for the observed anticancer properties. However, this appears quite particular because the other NHC complexes bearing gold did not induce any effect at all (see **L1AuCl**, **L1AuOAc** and **L2AuCl**, Table 1), but they possess different ligands. Some deductions can be done observing the chemical structures of the above‐mentioned gold‐based complexes; particularly, **L2AuOAc** differs from **L2AuCl** just for the counterion (acetate vs. chloride), from **L1AuCl** for the lacking of a ribose residue bound to the N9 position of the carbene ring and for the counterion (chloride), and from **L1AuOAc** just for the ribose substituent at the N9 position. Thus, it seems that the contemporaneous presence of the counterion acetate and ribose plays a critical role in maintaining the anticancer effect in NHC‐gold complexes. Additionally, the corresponding silver complexes **L1AgI**, **L2AgI** and, as said before, **L2AgOAc**, completely lacked any activity, with the only exception of **L1AgOAc** that, conversely, showed a low activity in all the three cancer cell types, without affecting the viability of normal breast cells at the same time. Regarding the NHC‐silver complexes, the inclusion of a glycine or a phenylalanine as counterions seem to produce a gain in the activity, as in **L1AgGly/L1AgPhe** and **L2AgGly/L2AgPhe** with respect to **L1AgI** and **L2AgI**, regardless of the presence of ribose as substituent. This strategy was successfully used in our previous published results, but with different NHC scaffolds [21]. However, in this case, the IC_50_ values of the glycinate/phenylalaninate derivatives did not reach the low micromolar range previously obtained, suggesting that this modification, by itself, is not sufficient for ameliorating the activity. This deduction is further reinforced by the observation that the activity of the analogues **L2AgGly** and **L2AgPhe**, bearing the counterions glycine or phenylalanine, was not improved in any cell line with respect to complex **L2AuOAc.** Summing up, **L2AuOAc** has been proved to be the best complex, amongst all, able to produce significant effects mostly against the triple negative MDA‐MB‐231 cells, and without affecting the viability of the normal counterpart, namely MCF‐10A cells.

**TABLE 1:** IC_50_ values of NHC‐gold and ‐silver complexes, expressed as μM. The means ± standard deviations are shown.

	MCF‐7	MDA‐MB‐231	HeLa	MCF‐10A
L1AgI	>200	>200	>200	>200
L1AgGly	73.8 ± 1.8	61.8 ± 1.3	48.7 ± 0.9	>200
L1AgOAc	73.6 ± 2.5	69.7 ± 1.3	111.2 ± 2.7	>200
L1AgPhe	97.04 ± 2.2	85.9 ± 1.7	>200	>200
L2AgI	>200	>200	>200	>200
L2AgGly	94.2 ± 1.6	80.3 ± 1.5	>200	>200
L2AgOAc	>200	>200	>200	>200
L2AgPhe	58.8 ± 0.5	51.8 ± 0.6	64.2 ± 0.8	>200
L1AuCl	>200	>200	>200	>200
L1AuOAc	>200	>200	>200	>200
L2AuCl	>200	>200	>200	>200
L2AuGly	193.1 ± 1.5	57.3 ± 1.1	190.3 ± 3.2	>200
L2AuOAc	42.9 ± 2.3	17.0 ± 0.3	49.0 ± 1.1	>200
L2AuPhe	195.5 ± 1.8	138.8 ± 2.6	189.7 ± 4.2	>200

### 2.5 Tunel Assay

**FIGURE 3:**
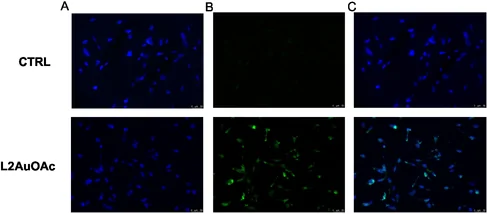
TUNEL assay. MDA‐MB‐231 breast cancer cells were treated with **L2AuOAc**, at the concentration equal to its IC_50_, or vehicle (CTRL) for 24 h. After the processing, images were visualized and acquired using a fluorescence microscope (20× magnification). Green fluorescence indicates nuclei of cells with fragmented DNA. Panels A, DAPI λ_ex/em_ 350 nm/460 nm. Panels B, CF488 A λ_ex/em_ 490 nm/515 nm. Panels C shows the merge.

Aiming at investigating whether **L2AuOAc** impacted the viability of MDA‐MB‐231 cells by damaging the nuclear DNA and, consequently, trigger cell death by apoptosis, a TUNEL assay was performed. Apoptosis is a biological process that removes, in a controlled way, damaged cells, without disturbing the near cells equilibrium [38, 39]. Cancer cells can proliferate without control, negatively impacting the cell homeostasis, and may escape from the normal senescence and death processes [40]. It becomes evident that apoptosis is also one of the key mechanisms for the elimination of cancer cells, and that the use of drugs able to induce apoptosis represent an important strategy for tumour therapy [41, 42]. Figure 3 shows that under **L2AuOAc** exposure, used at its IC_50_ value for 24 h, as detailed in the experimental section, a net green fluorescence is evident, indicating the presence of fragmented DNA (panel B). Panel C displays the overlay between panels B and A, the latter showing the DAPI nuclear staining, which demonstrates the damage of the nuclear DNA. Conversely, no fluorescence is visible in the control experiment, where cells were treated with the only solvent (DMSO), indicating the absence of nuclear DNA damage (panel B, CTRL). Several cytotoxic anticancer agents, also used in therapy, target and damage DNA [43, 44, 45] and it is widely accepted that the cellular response to nuclear DNA damage resides in the activation of checkpoint mechanisms that induces the cell cycle arrest or, ultimately, death, most commonly by apoptosis [46, 47]. As well in this case, the impact of **L2AuOAc** depends on its ability in damaging MDA‐MB‐231 cells DNA, amongst other possible targets, and push them to die by triggering the apoptotic mechanism.

### 2.6 Mitochondrial Membrane Potential (MMP) Evaluation

**FIGURE 4:**
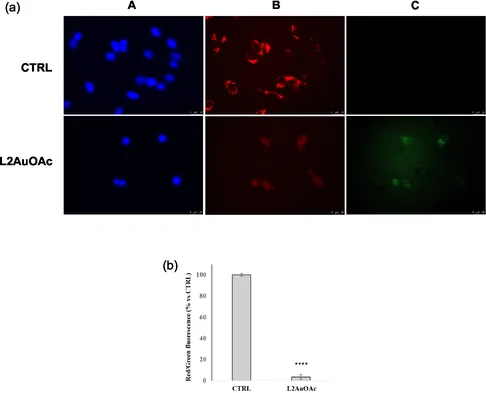
(a) Evaluation of the mitochondrial membrane potential (MMP) by the means of the JC‐1 fluorescent probe in MDA‐MB‐231 breast cancer cells, treated with DMSO (vehicle) or **L2AuOAc** (17 µM, 24 h). Representative fields are shown. Panels: (A) DAPI (λ_ex/em_ = 350/460 nm); (B) red fluorescence from JC‐1 aggregates (λ_ex/em_ = 644/665 nm); (C) green fluorescence from JC‐1 monomers (λ_ex/em_ = 490/515 nm). (b) Red‐to‐green fluorescence intensity ratio, percentage (%) versus CTRL. **** *p* < 0.0001, **L2AuOAc** versus CTRL.

Once demonstrated the ability in damaging the genomic DNA, exerted by **L2AuOAc** in MDA‐MB‐231 breast cancer cell, the Mitochondrial Membrane Potential (MMP or ΔΨm) was also evaluated by the means of the JC‐1 fluorescent probe. As it is known, mitochondria are dynamic organelles playing a major role in physiological functions, as energy generation, metabolic regulation, and signal transduction, but also responsible for several cellular processes importantly related to cancer onset and development [48]. Moreover, different NHC‐metal complexes have been demonstrated to disrupt the mitochondrial function, inducing Reactive Oxygen Species (ROS) production and cancer cell death by apoptosis [12]. Thus, MDA‐MB‐231 cells were treated with 17 µM of **L2AuOAc** or vehicle (DMSO, negative control) for 24 h, then incubated with the JC‐1 fluorescent probe, properly processed and observed. The outcomes are shown in Figure 4a and b, where it is possible to see that the DMSO‐treated cells exhibited a mitochondrial red fluorescence, which indicate a high MMP (CTRL, panel B, Figure 4a). Contrarily, the exposure to **L2AuOAc** produced a reduction of the red fluorescence and an increase of the green one, as visible in Figure 4a, **L2AuOAc**, panels B and C, respectively. It is known that JC‐1 reports the membrane potential ratiometrically, forming J‐aggregates that emits a red fluorescence at high ΔΨm and reverting to green emitting monomers upon depolarization [49]. Under the activation of the apoptotic mechanism that involves the mitochondrial compartment impairment, JC‐1 is present in the monomeric form. Figure 4b shows the calculated red‐to‐green fluorescence intensity ratio.

### 2.7 Intracellular ROS Production

**FIGURE 5:**
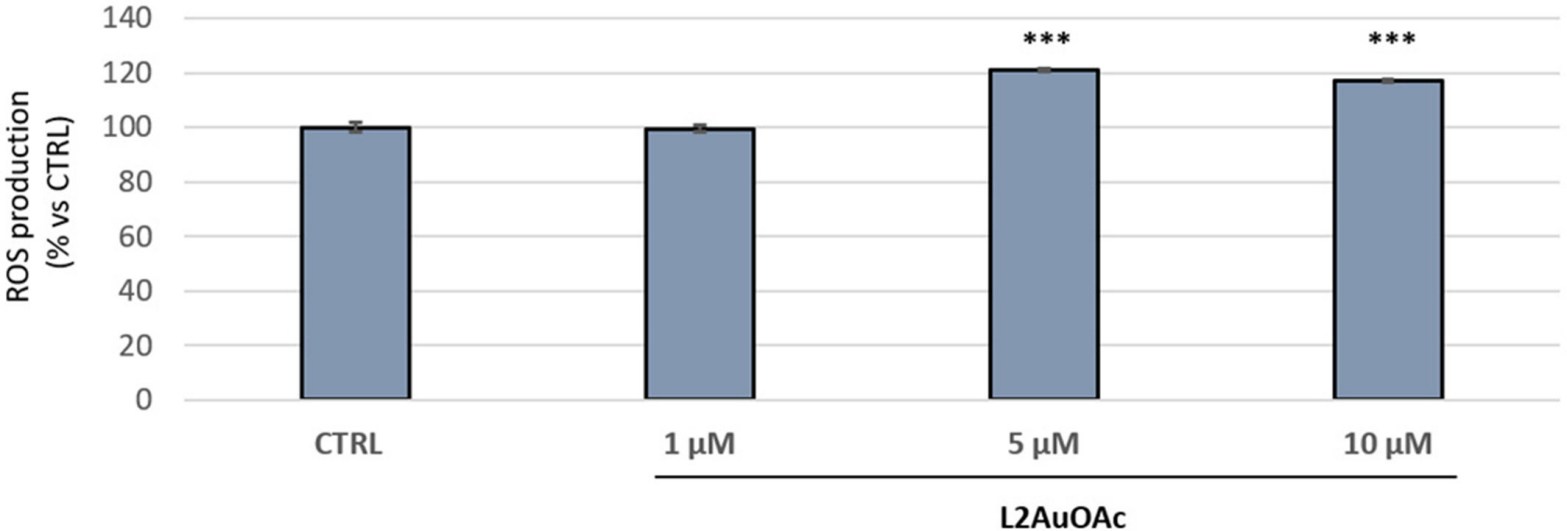
Reactive Oxygen Species (ROS) determination in MDA‐MB‐231 cells treated with the vehicle (DMSO, CTRL) or **L2AuOAc** at the indicated concentrations (1, 5 and 10 µM) for 24 h. *** *p* < 0.001, or not significant where not indicated, **L2AuOAc** versus CTRL.

The collapse of MMP is generally concomitant with the production of ROS, which is coupled with the antioxidant systems depletion. The latter can induce damaging effects on major cellular components, for instance DNA, proteins, and lipids, ultimately leading to the induction/potentiation of the apoptotic cell death [50]. In order to measure the ability of **L2AuOAc** in inducing the oxidative stress in MDA‐MB‐231 cells, we adopted the dihydro‐2^′^,7^′^‐dichlorofluorescein diacetate (H_2_DCF‐DA) fluorescent probe. Figure 5 shows the effect of the treatment with **L2AuOAc**, for 24 h, at 1, 5 and 10 µM. As it is possible to see, the highest effect has been recorded at 5 µM, with an increase of intracellular ROS levels of about 21%, compared to the DMSO‐treated cells (vehicle, CTRL). At the lowest concentration, no significant rise of intracellular ROS was detected, whereas the percentage remains quite unvaried at the highest dose used (10 µM) compared to the treatment with 5 µM of **L2AuOAc**. When the intracellular ROS levels exceed a critical threshold, different forms of programmed cell death can be activated and one of the most studied is apoptosis [51].

### 2.8 Caspases Activity Determination

**FIGURE 6:**
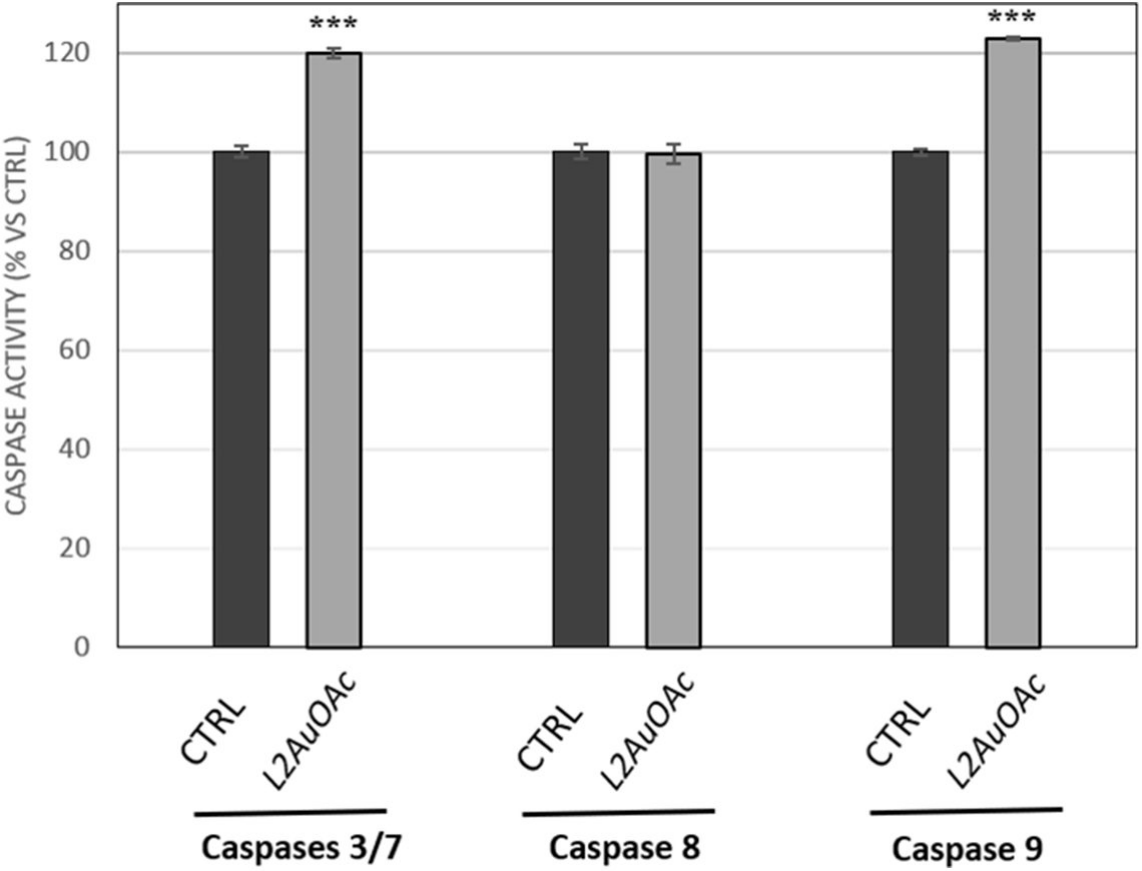
Caspases 3/7, 8 and 9 activity determination. MDA‐MB‐231 cells were treated with **L2AuOAc** (17 µM, 24 h), then the caspases activity was measured and reported as percentage versus the DMSO‐treated cells, used as control (CTRL). Bars represent the means ± SD of three different experiments. ***p < 0.001, or not significant where not indicated, **L2AuOAc** versus CTRL.

The previously reported results strongly suggest that the treatment with **L2AuOAc** induces MDA‐MB‐231 cells death by apoptosis, which represents one of the well‐documented‐characterized forms of regulated cell death. Apoptosis includes two major pathways, the intrinsic, or mitochondrial, and the extrinsic, or death receptor–mediated. Furthermore, ROS balance and oxidative signals are directly linked to the apoptosis triggering, since they are important regulators of the cell fate [51]. In any case, the activation of caspases is finely orchestrated in apoptosis, allowing the stepwise degradation of intracellular structures. In order to determine definitively whether MDA‐MB‐231 cells were undergoing to apoptosis after the exposure to **L2AuOAc**, at 17 µM for 24 h, the activity of the initiator caspases 8 and 9 and the executioner caspases 3/7 was detected, as detailed in the experimental section. Figure 6 resumes the obtained outcomes, indicating that the activity of caspases 9 and 3/7 was found increased, compared to the DMSO‐treated cells (vehicle). More in detail, a rise of the activity of about 20% was detected for caspases 3/7, which are usually cleaved and activated by the initiator caspase 9. The latter activity was also found risen of about 23%, whereas no significant difference was found for caspase 8 activity, whether compared to the vehicle‐treated cells (Figure 6). Taking together, these results strongly indicated that the intrinsic apoptotic pathway is taking place in the considered cell context and under **L2AuOAc** exposure.

### 2.9 Antiviral Activity

**FIGURE 7:**
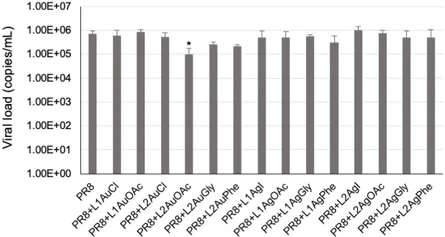
Viral load quantified in supernatants from BEAS‐2B cells infected with PR8 virus and treated with NHC‐metal complexes (10 μM) for 24 h by qRT‐PCR, using Hemagglutinin (HA)‐specific primers. Data are shown as mean ± SD, *p < 0.05.

**FIGURE 8:**
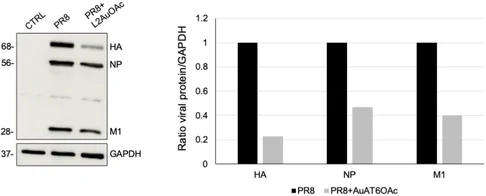
Western blot analysis of Influenza virus proteins in cell lysates of PR8‐infected BEAS‐2B cells treated and not‐treated with **L2AuOAc** (10 μM) for 24 h. GAPDH was used as loading control. In the graph on the right of the western blot images, densitometric analysis is shown, expressed as ratio of each viral protein to GAPDH.

**FIGURE 9:**
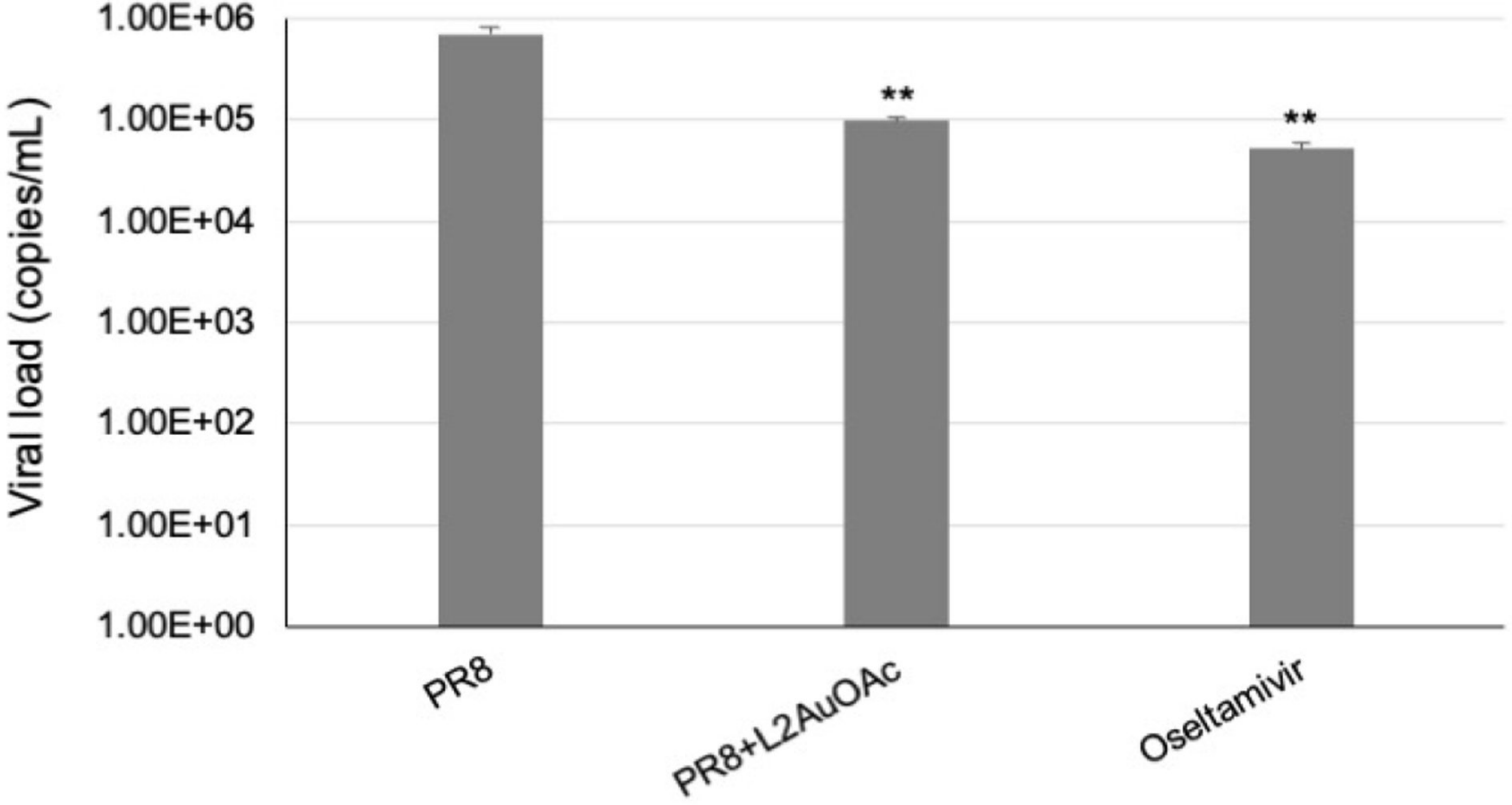
Viral load quantified in supernatants from BEAS‐2B cells infected with PR8 virus and treated with **L2AuOAc** (10 μM) or Oseltamivir carboxylate (1 μM) for 24h by qRT‐PCR, using HA‐specific primers. Data are shown as mean ± SD, **p < 0.001.

To evaluate the potential antiviral activity of the guanosine‐ and guanine‐derived *N*‐heterocyclic carbene silver(I) and gold(I) complexes, BEAS‐2B cells were infected with Influenza virus A/Puerto Rico/8/34 H1N1 (PR8) and treated with each compound at 10 μM for 24 h. The viral load was quantified in the supernatants by qRT‐PCR. As shown in Figure 7, the decrease in viral load, induced by some compounds, was significant in the case of treatment with **L2AuOAc.** In Figure 8, Western blot analysis with anti‐Influenza antibody of cell lysates of PR8‐infected BEAS‐2B cells treated and not‐treated with **L2AuOAc**, showed a lower expression of the main viral proteins, that is Hemagglutinin (HA), Nucleoprotein (NP) and Matrix Protein1 (M1), which therefore can hesitate in the reduced viral titer measured in the treated samples. Finally, the viral titration assay was repeated under the same conditions as before, using Oseltamivir carboxylate as a positive control. The result in Figure 9 showed a significant inhibition by **L2AuOAc** that was comparable to that obtained with Oseltamivir (in percentage, 86.2% vs. 92.7%).

The efficacy of **L2AuOAc,** at a not cytotoxic concentration, in reducing influenza virus replication in an in vitro infection model, represented by the non‐tumorigenic bronchial epithelial cell line BEAS‐2B, indicates antiviral activity, over the anticancer one. Moreover, the data indicates a marked antiviral activity approaching that of the neuraminidase inhibitor, Oseltamivir, in our experimental model. The antiviral effect of **L2AuOAc** could be exerted directly against the virus or on cellular pathways specifically activated during viral infection. Further studies may be aimed at investigating the underlying mechanisms.

## 3 Conclusion

In summary, this study reports an effective strategy for the design of novel NHC silver(I) and gold(I) using guanine and guanosine as scaffolds. The synthetic route successfully yielded a library of silver(I) and gold(I) complexes, with the presence of various counterions (iodide, acetate, and aminoacidate), aiming at providing a versatile method for tuning the lipophilicity and electronic properties of the metal center. Based on general coordination chemistry considerations, the iodide‐containing complexes are expected to exhibit a higher lipophilic character than the corresponding acetate and aminoacidate derivatives, whereas the latter are likely to display increased polarity due to the presence of oxygen‐ and nitrogen‐containing functional groups. From a structural‐activity perspective, the chemical data suggest that: first the nature of the metal center plays a crucial role, with gold(I) complexes consistently exhibiting superior biological activity compared with their silver(I) counterparts, in agreement with the well‐established pharmacological potential of gold(I) NHC complexes. Second, the nature of the nucleobase scaffold also appears to influence the biological response. Complexes incorporating the guanosine scaffold generally displayed improved activity compared with the corresponding guanine derivatives, suggesting that the ribose moiety may favor biological performance, possibly by modulating the overall polarity, molecular recognition, or cellular uptake of the complexes. Finally, the ancillary ligand significantly affected the biological profile. Although lipophilicity was not experimentally determined, the different counterions are expected to alter the physicochemical properties of the complexes, which may influence their interaction with biological targets and their intracellular distribution. Within the investigated series, the synergistic combination of a gold(I) center, the ribose moiety and the acetate counterion, as seen in the individuated lead, namely **L2AuOAc**, is pivotal for producing the best biological efficacy. Indeed, **L2AuOAc** was found particularly active and selective against the high aggressive MDA‐MB231 breast cancer cells, without interfering with the viability of the normal breast MCF‐10A cells. Further studies revealed that this complex was able to induce genomic DNA damage in MDA‐MB231 breast cancer cells, after 24 h of exposure, which pushes cancer cells to die by the intrinsic apoptotic mechanism. Furthermore, the same above mentioned complex possesses a notable antiviral activity, as demonstrated by the reduction of the A/Puerto Rico/8/34 H1N1 influenza virus replication in the adopted in vitro model, modulating the virus life cycle or cellular pathways activated during the viral infection, which specific mechanisms deserve to be further investigated. Overall, this report gives some hints about the synthesis of NHC derived from guanine and guanosine scaffolds and the easy “tune” of complexes properties, for instance by structural modifications or counterions change, for the precision drug design, and also providing useful lead(s) gifted with a dual biological activity, namely anticancer and antiviral. This is a pivotal field of study in medicinal chemistry, addressing two major global health challenges, namely cancer treatment resistance onset and the emergence of new viral threats. Of course, the most promising complex needs to be further developed in order to optimize the biological activity and individuate other targets that could be also regulated, together with in vivo studies that could disclose the therapeutic potential and/or limitations.

## 4 Experimental Section

All the chemicals and reagents were purchased from commercial suppliers (Sigma–Aldrich‐Merck, Milan, Italy) and used without further purification. All syntheses were carried out in a nitrogen atmosphere, using Schlenk techniques.

All newly synthesized complexes were characterized by ^1^H and ^13^C NMR spectroscopy, high‐resolution mass spectrometry (HRMS), and elemental analysis. The combined analytical data were consistent with the proposed structures and confirmed the identity and satisfactory purity of the isolated compounds.

Nuclear magnetic resonance (NMR) spectroscopy was carried out on a BRUKER AVANCE 400 spectrometer (400 MHz for ^1^H; 100 MHz for ^13^C) and BRUKER AM 300 spectrometer (300 MHz for ^1^H; 75 MHz for ^13^C). NMR samples were prepared using approximately 8 mg of sample and using DMSO‐d_6_ as solvent. The chemical shifts of the ^1^H NMR and ^13^C NMR spectra are given in ppm relative to the residual solvent peak of the deuterated solvent used (DMSO, ^1^H NMR 2.50 ppm, and ^13^C NMR 39.00 ppm). The multiplicities have been reported as: (s)inglet, (d)oublet, (t)riplet, unresolved (m)ultiplet, (o)verlapped, and (br)oad.

Elemental analyses were performed using a PerkinElmer 2400 CHN elemental analyzer.

MALDI‐MS mass spectra were achieved by Bruker SolariX XR Fourier transform ion cyclotron resonance mass spectrometer with a 7 T refrigerated actively shielded superconducting magnet.

### 4.1 Synthesis of 2‐amino‐7,9‐dimethyl‐1,9‐dihydro‐6H‐7l4‐purin‐6‐one iodide (PL1)

The reaction was carried out in a 50 mL two‐necked flask equipped with a magnetic stirrer and a reflux condenser. Guanine (1.00 g, 6.62 mmol) was dissolved in anhydrous *N*,*N*‐dimethylacetamide (DMA, 30 mL), followed by the addition of an excess of iodomethane (1.25 mL, 20 mmol). The reaction mixture was stirred for 24 h at room temperature. Subsequently, the mixture was transferred to a 250 mL flask and stirred for 1 h with 200 mL of diethyl ether, yielding a yellow oil. After the liquid phase was decanted, an additional 200 mL of Et_2_O was added, and the suspension was stirred for an additional 6 h. The final product was obtained as a white solid after crystallization from methanol [35]. Yield: 81%. ^1^H‐NMR (300 MHz, DMSO‐*d*_6_, 298 K) δ 11.6_2_ (s, 1H, NH), 9.1_4_ (s, 1H, NCHN), 7.1_5_ (br, 2H, NH_2_), 3.9_9_ (s, 3H, NCCH_3_), 3.6_9_ (s, 3H, OCCNCH_3_). ^13^C‐NMR (100 MHz, DMSO‐*d*_6_, 298 K) δ 155.5_0_ (CNH_2_), 153.5_0_ (C═O), 149.9_8_ (NC4═C5N), 138.5_3_ (NCHN), 107.0_6_ (NC4═C5N), 35.2_4_ (NCCH_3_), 31.2_5_ (OCCNCH_3_). MALDI‐MS (CH_3_OH) m/z: 180.08799 Da attributable to [C_7_H_10_N_5_O]^+^.

### 4.2 Synthesis of 2‐amino‐9‐((2S,5S)‐3,4‐dihydroxy‐5‐(2‐hydroxyethyl)tetrahydrofuran‐2‐yl)‐7‐methyl‐1,9‐dihydro‐6H‐7l4‐purin‐6‐one iodide (PL2)

In a 25 mL two‐necked round‐bottom flask equipped with a magnetic stirrer and a reflux condenser, guanosine (0.500 g, 0.176 mmol) was dissolved in 9.0 mL of anhydrous *N*,*N*‐dimethylacetamide (DMA). Subsequently, iodomethane (0.333 mL, 5.35 mmol) was added to the solution. The reaction mixture was stirred at room temperature for 24 h. The mixture was then transferred into a 250 mL round‐bottom flask and stirred with 200 mL of diethyl ether (Et_2_O) for 1 h to induce precipitation. After the liquid phase was decanted, an additional 200 mL of Et_2_O was added. The resulting suspension was stirred for a further 6 h. The crude product was collected and purified via recrystallization from methanol, yielding the title compound as a white solid. Yield: 89%. ^1^H‐NMR (300 MHz, DMSO‐*d*_6_, 298 K) δ 11.7_4_ (s, br, 1H, NH), 9.3_4_ (s, 1H, NCHN) 7.2_2_ (s, br, 2H, NH_2_), 5.8_4_ (d, 1H, NCH), 5.6_6_ (d, 1H, CHOH), 5.3_5_ (d, 1H, CHOH), 5.1_3_ (t, 1H, CH_2_OH), 4.3_7_ (d, 1H, CHOH), 4.1_6_ (m, 1H, CHOH), 4.0_1_ (s br, 4H, CHCH_2_OH + NCH_3_), 3.7_4_−3.6_3_ (m, 2H, CH_2_OH). ^13^C‐NMR (75 MHz, DMSO‐*d*_6_, 298 K) δ: 155.6_1_ (CNH_2_), 153.4_5_ (C═O), 149.1_3_ (NC4═C5N), 136.3_7_ (NCHN), 107.5_5_ (NC4═C5N), 88.6_8_ (NCH), 85.6_3_ (CHCH_2_), 74.2_2_ (CHOH), 69.2_7_ (CHOH), 60.3_7_ (CH_2_OH), 35.7_2_ (NCH_3_). MALDI‐MS: (CH_3_OH) m/z: 298.11460 Da attributable to [C_11_H_16_N_5_O_5_]^+^.

### 4.3 Synthesis of (2‐amino‐7,9‐dimethyl‐6‐oxo‐6,7,8,9‐tetrahydro‐1H‐purin‐8‐yl)silver(II) iodide (L1AgI)

PL1 salt (0.500 g, 1.63 mmol) was placed in a 100 mL two‐necked round‐bottom flask and dissolved in 60 mL of anhydrous methanol. Once dissolution was complete, aided by magnetic stirring, silver(I) oxide (0.226 g, 0.977 mmol) and molecular sieves were added to the solution. The reaction mixture was stirred at room temperature for 3 h. The final product was recovered by filtration through a pad of Celite to remove inorganic residues, followed by the removal of the solvent under reduced pressure. Yield: 40%. ^1^H‐NMR (300 MHz, DMSO‐*d*_6_, 298 K) δ 4.0_1_ (s, 3H, OCCNCH_3_), 3.7_8_ (s, 3H, NCCH_3_). ^13^C‐NMR (100 MHz, DMSO‐*d*_6_, 298 K) δ 177.8_3_ (NCN), 155.7_6_ (CNH_2_), 154.5_4_ (C═O), 150.2_3_ (NC4═C5N), 108.3_8_ (NC4═C5N), 36.3_7_ (NCCH_3_), 31.7_9_ (OCCNCH_3_). Elemental analysis calcd for C_7_H_9_AgIN_5_O (%): C 20.31, H 2.19, N 16.92; found (%): C 20.28, H 2.26, N 16.99. MALDI‐MS: (CH_3_OH) m/z: 467.07141 Da attributable to [C_14_H_20_AgN_10_O_2_] ^+^.

### 4.4 Synthesis of (2‐amino‐9‐((2S,5S)‐3,4‐dihydroxy‐5‐(2‐hydroxyethyl)tetrahydrofuran‐2‐yl)‐7‐methyl‐6‐oxo‐6,7,8,9‐tetrahydro‐1H‐purin‐8‐yl)silver(II) iodide (L2AgI)

In a 50 mL two‐necked round‐bottom flask equipped with a magnetic stirrer and a reflux condenser, the **PL2** salt (0.160 g, 0.376 mmol) was dissolved in 15 mL of anhydrous methanol. Subsequently, silver(I) oxide (0.0520 g, 0.226 mmol) and molecular sieves were added. The reaction mixture was stirred at room temperature for 3 h. The product was isolated by filtration through a Celite pad, followed by the removal of the solvent in vacuo. Yield: 43%. ^1^H‐NMR (300 MHz, DMSO‐*d*_6_, 298 K) δ 8.9_9_ (s, 2H, NH_2_), 5.8_0_ (d, 1H, NCH), 5.6_9_ (br, 3H, OH), 4.4_7_ (d, 1H, CHOH), 4.1_3_ (m, 1H, CHOH), 4.0_0_ (s br, 4H, CHCH_2_OH + NCH_3_), 3.6_9_−3.5_9_ (m, 2H, CH_2_OH). ^13^C‐NMR (100 MHz, DMSO‐*d*_6_, 298 K) δ: 173.5_6_ (NCN), 155.0*1* (CNH_2_), 152.6_3_ (C═O), 148.1_9_ (NC4═C5N), 108.5_5_ (NC4═C5N), 90.6 (NCH), 88.6 (CHCH_2_), 75.2 (CH_2_OH), 70.2 (CHOH), 63.3_4_ (CH_2_OH), 35.8_5_ (NCH_3_). Elemental analysis calcd for C_11_H_15_AgIN_5_O_5_ (%): C 24.83, H 2.84, N 13.16; found (%): C 24.63, H 2.79, N 13.03. MALDI‐MS: (CH_3_OH) m/z: Da 617.20978 attributable to [C_18_H_25_AgN_10_O_8_]^+^.

### 4.5 Synthesis of acetyl(2‐amino‐7,9‐dimethyl‐6‐oxo‐6,7,8,9‐tetrahydro‐1H‐purin‐8‐yl)(oxo)silver (L1AgOAc)

In a 50 mL two‐necked flask equipped with a magnetic stirrer and a reflux condenser, AgL1I (0.102 g, 0.246 mmol) was dissolved in 10 mL of dry methanol. Subsequently, a solution of AgOAc in acetonitrile (0.041 g/7 mL) was added. The reaction mixture was stirred at room temperature for 24 h. The resulting product was collected via filtration, washed with acetonitrile, and dried under vacuum to remove any residual solvent. Yield: 20%. ^1^H‐NMR (300 MHz, DMSO‐*d*_6_, 298 K) δ 4.0_7_ (s, 3H, OCCNCH_3_), 3.8_0_ (s, 3H, NCCH_3_), 1.7_5_ (s, 3H, OCOCH_3_). ^13^C‐NMR (100 MHz, DMSO‐*d*_6_, 298 K) δ 175.2_3_ (OC*═*OCH_3_), 174.8_5_ (NCN), 155.5_8_ (CNH_2_), 154.1_8_ (C═ONH), 149.2_7_ (NC4═C5N), 107.3_9_ (NC4═C5N), 36.3_3_ (OCCNCH_3_), 31.7_5_ (NCCH_3_), 22.2_4_ (OCOCH_3_). Elemental analysis calcd for C_9_H_12_AgIN_5_O_3_ (%): C 31.23, H 3.49, N 20.24; found (%): C 31.15, H 3.58, N 20.19. MALDI‐MS: (CH_3_OH) m/z: 465.06793 Da attributable to [C_14_H_18_AgN_10_O_2_]^+^.

### 4.6 Synthesis of acetyl(2‐amino‐9‐((2S,5S)‐3,4‐dihydroxy‐5‐(2‐hydroxyethyl)tetrahydrofuran‐2‐yl)‐7‐methyl‐6‐oxo‐6,7,8,9‐tetrahydro‐1H‐purin‐8‐yl)(oxo)silver (L2AgOAc)

In a 200 mL two‐necked round‐bottom flask equipped with a magnetic stirrer and a reflux condenser, the salt PL2 (0.500 g, 1.17 mmol) was dissolved in 45 mL of dry methanol. Subsequently, a solution of AgOAc (0.393 g) in acetonitrile (35 mL) was added. The reaction mixture was stirred for 24 h at room temperature. The resulting product was collected via filtration, washed with acetonitrile (3 × 20 mL), and isolated by solvent evaporation. Yield: 25%.

^1^H‐NMR (300 MHz, DMSO‐*d*_6_, 298 K) δ 5.8_0_ (d, 1H, NCH), 5.6_9_ (d, 1H, CHOH), 4.4_7_ (d, 1H, CHOH), 4.1_3_ (m, 1H, CHOH), 4.0_0_ (s br, 4H, CHCH_2_OH + NCH_3_), 3.6_9_−3.5_9_ (m, 2H, CH_2_OH), 1.8_5_ (s, 3H, OCOCH_3_). ^13^C‐NMR (100 MHz, DMSO‐*d*_6_, 298 K) δ 175.2_3_ (OC*═*OCH_3_), 169.8_6_ (NCN), 155.6_4_ (CNH_2_), 152.5_2_ (C═ONH), 148.0_1_ (NC═C5N), 109.9_4_ (NC4═C5N), 90.7_4_ (NCH), 88.5_2_ (CHCH_2_), 75.0_9_ (CHOH), 70.8_8_ (CHOH), 63.3_7_ (CH_2_OH), 35.5_2_ (NCH_3_), 23.8_5_ (OCOCH_3_). Elemental analysis calcd for C_13_H_18_AgN_5_O_7_ (%): C 33.64, H 3.91, N 15.09; found (%): C 33.51, H 3.99, N 15.00. MALDI‐MS: m/z: 701.842181 Da attributable to [C_22_H_31_AgN_10_O_10_]^+^.

### 4.7 Synthesis of Complexes with Amino Acid Derivatives

Boc‐protected amino acid silver salts (Boc‐Gly‐Ag and Boc‐L‐Phe‍‐‍Ag) were synthesized according to literature procedures [52, 53].

### 4.8 General Proc*edure*

The synthesis was performed in a two‐necked round‐bottom flask equipped with a magnetic stirrer and a reflux condenser. The starting silver complex (**LAgI**) was dissolved in dry methanol. In a separate flask, the silver‐amino acid salt (Boc‐AA‐Ag) was solubilized in acetonitrile via sonication. This solution was then added dropwise to the reaction flask. The mixture was allowed to stir at room temperature for 24 h.

The resulting suspension was filtered through a Celite pad to remove insoluble residues. The filtrate was then concentrated to dryness under reduced pressure (in vacuo) to yield the final product.

### 4.9 Synthesis of (2‐amino‐7,9‐dimethyl‐6‐oxo‐6,7,8,9‐tetrahydro‐1H‐purin‐8‐yl)(oxo)((2‐oxoethyl)amino)silver (L1AgGly)

**L1AgI** (0.050 g, 1.21 × 10^−4^ mol) was dissolved in 20 mL of dry methanol.

Boc‐Gly‐Ag (0.040 g, 1.45 × 10^−4^ mol) was dissolved in 40 mL of acetonitrile.

Yield: 60% ^1^H‐NMR (300 MHz, DMSO‐*d*_6_, 298 K) δ 7.1_0_ (br, 1H, CH_2_NH), 3.8_7_ (s, 3H, OCCNCH_3_), 3.5_5_ (d, 2H, CH_2_), 3.5_1_ (s, 3H, NCCH_3_), 1.3_8_ (s, 9H, C(CH_3_)_3_). ^13^C‐NMR (100 MHz, DMSO‐*d*_6_, 298 K) δ 174.1_1_ (AgOC*═*O), 171.4_6_ (NCN), 155.3_2_ (NHC*═*OO), 154.0_7_ (CNH_2_), 147.7_3_ (CCONH), 140.4_8_ (NC4═C5N), 110.5_3_ (NC4═C5N), 77.5_5_ (C(CH_3_)_3_), 43.4_2_ (CH_2_NH), 32.7_8_ (OCCNCH_3_), 30.6_4_ (NCCH_3_), 28.2_3_ (C(CH_3_)_3_). Elemental analysis calcd for C_14_H_21_AgN_6_O_5_ (%): C 36.46, H 4.59, N 18.22; found (%): C 36.37, H 4.69, N 18.11. MALDI‐MS: (CH_3_OH) m/z: 467.04102 Da attributable to [C_14_H_19_AgN_10_O_2_] ^+^.

### 4.10 Synthesis of (2‐amino‐9‐((2S,5S)‐3,4‐dihydroxy‐5‐(2‐hydroxyethyl)tetrahydrofuran‐2‐yl)‐7‐methyl‐6‐oxo‐6,7,8,9‐tetrahydro‐1H‐purin‐8‐yl)(oxo)((2‐oxo‐2l3‐ethyl)amino)silver (L2AgGly)

**L2AgI** (0.100 g, 1.88 × 10^−4^ mol) was dissolved in 25 mL of dry methanol.

Boc‐Gly‐Ag (0.064 g, 2.26 × 10^−4^ mol) was dissolved in 40 mL of acetonitrile.

Yield: 70%. ^1^H‐NMR (300 MHz, DMSO‐*d*_6_, 298 K) δ 7.7_6_ (br, 2H, NH_2_), 6.3_5_ (br, 1H, NH), 5.8_0_ (d, 1H, NCH), 4.47 (d, 1H, CHOH), 4.1_3_ (m, 1H, CHOH), 3.9_8_ (s br, 4H, CHCH_2_OH + NCH_3_), 3.6_9_−3.5_9_ (m, 2H, CH_2_OH), 3.6_4_−3.5_5_ (m, 2H, CH_2_NH) 1.3_8_ (s, 9H, C(CH_3_)_3_). ^13^C‐NMR (100 MHz, DMSO‐*d*_6_, 298 K) δ 174.1_6_ (AgOC*═*O), 170.5_4_ (NCN), 159.3_3_ (NHC═OO), 157.7_2_ (CNH_2_), 155.6_6_ (C*═*ONH), 148.1_9_ (NC4═C5N), 108.5_6_ (NC4═C5N), 90.6_8_ (NCH), 88.6_7_ (CHCH_2_), 77.5_4_ (C(CH_3_)_3_), 75.2_3_ (CH_2_OH), 70.2_8_ (CHOH), 63.3_7_ (CH_2_OH), 43.5_5_ (CH_2_NH), 35.8_9_ (NCH_3_), 28.2_7_ (C(CH_3_)_3_). Elemental analysis calcd for C_18_H_27_AgN_6_O_9_ (%): C 37.32, H 4.70, N 14.51; found (%): C 37.47, H 4.87, N 14.39. MALDI‐MS: (CH_3_OH) m/z: 703.11822 Da attributable to [C_22_H_31_AgN_10_O_10_] ^+^.

### 4.11 Synthesis of (2‐amino‐7,9‐dimethyl‐6‐‍‌‍oxo‐6,7,8,9‐tetrahydro‐1H‐purin‐8‐yl)(((Tert‐­Butoxycarbonyl)phenylalanyl)oxy)silver (L1AgPhe)

**L1AgI** (0.101 g, 2.43  × 10^−4^ mol) was dissolved in 40 mL of dry methanol.

Boc‐Phe‐Ag (0.108 g, 2.92 × 10^−4^ mol) was dissolved in 80 mL of acetonitrile.action was carried ou

Yield: 67%. ^1^H‐NMR (400 MHz, DMSO‐*d*_6_, 298 K) δ 7.1_6_ (m, 5H, aromatics), 6.8_2_ (br, 2H, CNH_2_), 6.3_0_ (br, 1H, CHNHCO), 4.0_2_ (s, 1H, CHNH), 3.8_6_ (s, 3H, OCCNCH_3_), 3.4_8_ (s, 3H, NCCH_3_), 3.0_6_−2.8_3_ (m, 2H, CH_2_Ph), 1.3_4_ (s, 9H, C(CH_3_)_3_). ^13^C‐NMR (100 MHz, DMSO‐*d*_6_, 298 K) δ 174.5_4_ (AgOC*═*O), 172.8_3_ (NCN), 155.3_2_ (NHC*═*OO), 154.0_1_ (CNH_2_), 147.7_8_ (CCONH), 140.4_9_ (NC4═C5N), 138.4_7_, 130.3_8_, 129.3_5_, 128.8_4_, 126.8_9_ (aromatic carbons), 109._7_ (NC4═C5N), 78.5_4_ (C(CH_3_)_3_), 56.7_3_ (COCHNH), 38.0_4_ (CH_2_Ph), 33.7_7_ (OCCNCH_3_), 31.5_4_ (NCCH_3_), 29.2_1_ (C(CH_3_)_3_). Elemental analysis calcd for C_21_H_27_AgN_6_O_5_ (%): C 45.75, H 4.94, N 15.24; found (%): C 45.58, H 5.13, N 15.39. MALDI‐MS: (CH_3_OH) m/z: 467.06525 Da attributable to [C_14_H_19_AgN_10_O_2_] ^+^.

### 4.12 Synthesis of (2‐amino‐9‐((2S,5S)‐3,4‐dihydroxy‐5‐(2‐hydroxyethyl)tetrahydrofuran‐2‐yl)‐7‐methyl‐6‐oxo‐6,7,8,9‐tetrahydro‐1H‐purin‐8‐yl)(oxo)(((S)‐1‐oxo‐3‐phenyl‐1l3‐propan‐2‐yl)amino)silver (L2AgPhe)

**L1AgI** (0.100 g, 1.88 × 10^−4^ mol) was dissolved in 25 mL of dry methanol.

Boc‐Phe‐Ag (0.084 g, 2.26 ×10^−4^ mol) was dissolved in 40 mL of acetonitrile.

Yield: 75%

^1^H‐NMR (300 MHz, DMSO‐*d*_6_, 298 K) δ 7.2_0_ (m, 5H, aromatics), 6.8_9_ (s, br, 2H, NH_2_), 6.2_5_ (s, br, 1H, NH), 5.7_6_ (d, 1H, NCH), 4.4_7_ (d, 1H, CHOH), 4.1_3_ (m, 2H, CHCH_2_Ph+CHOH), 3.8_5_ (s br, 4H, CHCH_2_OH + NCH_3_), 3.6_9_−3.5_9_ (m, 2H, CH_2_OH), 3.0_6_−2.8_3_ (m, 2H, CH_2_Ph), 1.3_4_ (s, 9H, C(CH_3_)_3_).

^13^C‐NMR (100 MHz, DMSO‐*d*_6_, 298 K) δ 174.5_3_ (AgOC*═*O), 171.0_7_ (NCN), 157.3_4_ (NHC═OO), 156.7_3_ (CNH_2_), 155.0_3_ (CONH), 148.1_2_ (NC4═C5N), 139.1_6_, 130.3_8_, 129.3_5_, 128.8_5_, 126.8_7_ (aromatic carbons), 108.5_2_ (NC4═C5N), 90.6_4_ (NCH), 88.6_3_ (CHCH_2_), 78.5_2_ (C(CH_3_)_3_), 75.2_5_ (CH_2_OH), 70.2_6_ (CHOH), 63.3_2_ (CH_2_OH), 56.7_1_ (CHNH), 38.0_2_ (CH_2_Ph), 35.8_6_ (NCH_3_) 29.2_8_ (C(CH_3_)_3_). Elemental analysis calcd for C_25_H_33_AgN_6_O_9_ (%): C 44.85, H 4.97, N 12.55; found (%): C 44.73, H 5.02, N 12.21. MALDI‐MS: (CH_3_OH) m/z: 701.119768 Da attributable to [C_22_H_30_AgN_10_O_10_] ^+^.

### 4.13 Synthesis of Gold(I) Complexes

#### 4.13.1 Synthesis of (2‐amino‐7,9‐dimethyl‐6‐oxo‐1,6,7,9‐tetrahydro‐8H‐purin‐8‐ylidene)gold(I) chloride (L1AuCl)

The reaction was carried out in a 50 mL two‐neck round‐bottom flask equipped with a magnetic stirrer and a condenser. **L1AgI** (0.113 g, 2.72 × 10^−4^ mol) was dissolved in 37 mL of dry methanol, and subsequently the gold precursor (Me)_2_SAuCl (0.080 g, 2.72 × 10^−4^ mol) was added. The reaction mixture was stirred at room temperature for 24 h. After the indicated time, the product was isolated by removal of the solvent under reduced pressure, followed by washing with dichloromethane and methanol. Yield: 45%.

^1^H‐NMR (400 MHz, DMSO‐*d*_6_, 298 K) δ 3.9_5_ (s, 3H, OCCNCH_3_), 3.4_8_ (s, 3H, s, 3H, NCCH_3_). ^13^C‐NMR (75 MHz, DMSO‐*d*_6_, 298 K) δ 167.8_2_ (NCN), 155.7_6_ (CNH_2_), 154.4_3_ (C═O), 150.2_3_ (NC4═C5N), 108.3_3_ (NC4═C5N), 36.3_2_ (OCCNCH_3_), 31.7_8_ (NCCH_3_). Elemental analysis calcd for C_7_H_9_AuClN_5_O (%): C 20.43, H 2.20, N 17.02; found (%): C 20.56, H 2.36, N 17.21. MALDI‐MS: m/z: 555.12657 Da attributable to [C_14_H_19_AuN_10_O_2_]^+^.

#### 4.13.2 Synthesis of (2‐amino‐9‐((2S,5S)‐3,4‐dihydroxy‐5‐(2‐hydroxyethyl)tetrahydrofuran‐2‐yl)‐7‐methyl‐6‐oxo‐6,7,8,9‐tetrahydro‐1H‐purin‐8‐yl)gold(I) chloride (L2AuCl)

The reaction was carried out in a 100 mL two‐neck round‐bottom flask equipped with a magnetic stirrer and a condenser. Under an inert nitrogen atmosphere, **L2AgI** (0.058 g, 1.09 × 10^−4^ mol) was dissolved in 60 mL of dry methanol, followed by the addition of the gold precursor (Me)_2_SAuCl (0.032 g, 1.09 × 10^−4^ mol). The reaction mixture was stirred at room temperature for 24 h. After the indicated time, the product was isolated by removal of the solvent under reduced pressure and subsequently washed with dichloromethane and methanol. Yield: 55%. ^1^H‐NMR (300 MHz, DMSO‐*d*_6_, 298 K) δ 5.8_7_ (d, 1H, NCH), 4.5_7_ (d, 1H, CHOH), 4.1_0_ (m, 1H, CHOH), 3.9_8_ (s br, 4H, CHCH_2_OH + NCH_3_), 3.6_9_−3.5_9_ (m, 2H, CH_2_OH). ^13^C‐NMR (100 MHz, DMSO‐*d*_6_, 298 K) δ 164.5_1_ (NCN), 155.7_2_ (CNH_2_), 152.5_7_ (C═O), 148.0_3_ (NC4═C5N), 108.5_2_ (NC4═C5N), 90.6_7_ (NCH), 88.6_9_ (CHCH_2_), 75.2_2_ (CHOH), 70.2_5_ (CHOH), 63.3_3_ (CH_2_OH), 35.8_6_ (NCH_3_). Elemental analysis calcd for C_11_H_15_AuClN_5_O_5_ (%): C 24.94, H 2.85, N 13.22; found (%): C 25.06, H 2.59, N 13.10. MALDI‐MS: (CH_3_OH) m/z: 791.17669 Da attributable to [C_22_H_30_AuN_10_O_10_] ^+^.

#### 4.13.3 Synthesis of acetyl(2‐amino‐7,9‐dimethyl‐6‐oxo‐6,7,8,9‐tetrahydro‐1H‐purin‐8‐yl)(oxo)gold(I) (L1AuOAc)

The reaction was carried out in a 50 mL two‐neck round‐bottom flask equipped with a magnetic stirrer and a condenser. **L1AuCl** (0.038 g, 9.23 × 10^−5^ mol) was dissolved in 20 mL of dry methanol, followed by the addition of silver acetate (AgOAc, 0.0185 g, 1.11 × 10^−4^ mol). The reaction mixture was heated to 60 °C and stirred for 24 h. After this time, the reaction product was isolated by filtration and removal of the solvent under reduced pressure. Yield: 88%.

^1^H‐NMR (300 MHz, DMSO‐*d*_6_, 298 K) δ 6.9_0_ (br, 2H, NH_2_), 3.9_5_ (s, 3H, OCCNCH_3_), 3.4_8_ (s, 3H, NCCH_3_), 1.8_4_ (s, 3H, OCOCH_3_). ^13^C‐NMR (100 MHz, DMSO‐_d6_, 298 K) δ 174.5_3_ (OC*═*OCH_3_), 161.8_6_ (NCN), 154.5_8_ (CNH_2_), 151.3_2_ (C═ONH), 147.7_6_ (NC4 ═C5N), 110.8_8_ (NC4═C5N) 32.8_4_ (OCCNCH_3_), 30.6_3_ (NCCH_3_), 23.8_7_ (OCOCH_3_). Elemental analysis calcd for C_9_H_12_AuN_5_O_3_ (%): C 24.84, H 2.78, N 16.09; found (%): C 24.89, H 2.98, N 16.23. MALDI‐MS: (CH_3_OH) m/z: 555.12657 Da attributable to [C_14_H_19_AuN_10_O_2_] ^+^.

#### 4.13.4 Synthesis of acetyl(2‐amino‐9‐((2S,5S)‐3,4‐dihydroxy‐5‐(2‐hydroxyethyl)tetrahydrofuran‐2‐yl)‐7‐methyl‐6‐oxo‐6,7,8,9‐tetrahydro‐1H‐purin‐8‐yl)(oxo)gold(I) (L2AuOAc)

The reaction was carried out in a 100 mL two‐neck round‐bottom flask equipped with a magnetic stirrer and a condenser. The gold complex **L2AuCl** (0.069 g, 1.30× 10^−4^ mol) was dissolved in 40 mL of dry methanol, followed by the addition of silver acetate (AgOAc, 0.026 g, 1.56 × 10^−4^ mol). The reaction mixture was stirred at 60 °C for 24 h. The product was then isolated by filtration and removal of the solvent under reduced pressure. Yield: 85%.^1^H‐NMR (300 MHz, DMSO‐*d*_6_, 298 K) δ 5.8_5_ (d, 1H, NCH), 4.6_0_ (d, 1H, CHOH), 4.4_9_ (m, 1H, CHOH), 4.1_0_ (o s, 5H, CHOH + CHCH_2_OH + NCH_3_), 3.7_5_−3.5_9_ (m, 2H, CH_2_OH), 1.8_4_ (s, 3H, COCH_3_). ^13^C‐NMR (100 MHz, DMSO‐*d*_6_, 2═C5N), 91.6_8_ (NCH), 88.6_4_ (CHCH_2_), 75.2_2_ (CHOH), 72.6_8_ (CHOH), 63.3_5_ (CH_2_OH), 36.3_6_ (NCH_3_) 23.8_5_ (OCOCH_3_). Elemental analysis calcd for C_13_H_18_AuN_5_O_7_ (%): C 28.22, H 3.28, N 12.66; found (%): C 28.11, H 3.10, N 12.69. MALDI‐MS: (CH_3_OH) m/z: 791.17739 Da attributable to [C_22_H_30_AuN_10_O_10_] ^+^.

#### 4.13.5 Synthesis of (2‐amino‐9‐((2S,5S)‐3,4‐dihydroxy‐5‐(2‐hydroxyethyl)tetrahydrofuran‐2‐yl)‐7‐methyl‐6‐oxo‐6,7,8,9‐tetrahydro‐1H‐purin‐8‐yl)(oxo)((2‐oxo‐2l3‐ethyl)amino)gold(I) (L2AuGly)

The synthesis was performed in a two‐necked round‐bottom flask equipped with a magnetic stirrer and a reflux condenser. The starting gold complex (**L2AuCl**, 0.051 g, 9.62 × 10^−5^ mol) was dissolved in 10 mL of dry methanol. In a separate flask, the silver‐amino acid salt (BocGlyAg, 0.029 g, 1.06 × 10^−4^ mol) was solubilized in 11 mL of acetonitrile via sonication. This solution was then added dropwise to the reaction flask. The mixture was allowed to stir at room temperature for 24 h. The resulting suspension was filtered through a Celite pad to remove insoluble residues. The filtrate was then concentrated to dryness under reduced pressure (in vacuo) to yield the final product. Yield: 65%. ^1^H‐NMR (300 MHz, DMSO‐*d*_6_, 298 K) δ 7.2_4_ (br, 2H, NH_2_), 6.4_3_ (br, 1H, NH), 5.8_1_ (d, 1H, NCH), 4.3_9_ (d, 1H, CHOH), 4.1_4_ (m, 1H, CHOH), 4.0_0_ (s br, 4H, CHCH_2_OH + NCH_3_), 3.6_7_−3.5_6_ (m, 2H, CH_2_OH), 3.5_4_−3.4_2_ (m, 2H, CH_2_NH) 1.3_6_ (s, 9H, C(CH_3_)_3_). ^13^C‐NMR (100 MHz, DMSO‐*d*_6_, 298 K) δ 173.4_8_ (AgOC*═*O), 158.5_8_ (NCN), 156.7_3_ (NHC═OO), 155.9_1_ (CNH_2_), 149.8_1_ (CCONH), 135.7_2_ (NC4═C5N), 107.8_7_ (NC4═C5N), 88.9_2_ (NCH), 86.0_6_ (CHCH_2_), 78.0_8_ (C(CH_3_)_3_), 74.6_1_ (CH_2_OH), 69.7_4_ (CHOH), 60.8_4_ (CH_2_OH), 43.7_6_ (CH_2_NH), 36.0_1_ (NCH_3_), 28.6_4_ (C(CH_3_)_3_). Elemental analysis calcd for C_18_H_27_AuN_6_O_9_ (%): C 32.34, H 4.07, N 12.57; found (%): C 32.54, H 4.19, N 12.45. MALDI‐MS: (CH_3_OH) m/z: 629.21990 Da attributable to [C_18_H_20_N_6_O_7_Au] ^+^.

#### 4.13.6 Synthesis of (2‐amino‐9‐((2S,5S)‐3,4‐dihydroxy‐5‐(2‐hydroxyethyl)tetrahydrofuran‐2‐yl)‐7‐methyl‐6‐oxo‐6,7,8,9‐tetrahydro‐1H‐purin‐8‐yl)(oxo)(((S)‐1‐oxo‐3‐phenyl‐1l3‐propan‐2‐yl)amino)gold(I) (L2AuPhe)

The reaction was performed in a two‐necked round‐bottom flask equipped with a reflux condenser and a magnetic stirrer. To a solution of the gold complex **L2AuCl** (0.051 g, 9.62× 10^−5^ mol) in dry methanol (10 mL), a sonicated solution of BocPheAg (0.039 g, 1.06 × 10^−4^ mol) in acetonitrile (11 mL) was added slowly. The reaction was maintained at room temperature for 24 h. Following this, the suspension was passed through a Celite pad to eliminate insoluble by‐products. Evaporation of the volatiles under reduced pressure yielded the crude product. Yield: 52%. ^1^H‐NMR (300 MHz, DMSO‐*d*_6_, 298 K) δ 7.1_9_ (m, 5H, aromatics), 6.2_9_ (br, 2H, NH_2_), 5.8_3_ (d, 1H, NCH), 4.3_9_ (m, 2H, NCH + CHOH), 4.0_1_ (m, 2H, CHCH_2_Ph+CHOH), 3.9_2_ (s br, 4H, CHCH_2_OH + NCH_3_), 3.6_9_−3.6_1_ (m, 2H, CH_2_OH), 3.0_4_−2.8_8_ (m, 2H, CH_2_Ph), 1.3_3_ (s, 9H, C(CH_3_)_3_. ^13^C‐NMR (100 MHz, DMSO‐*d*_6_, 298 K) δ 175.1_4_ (AgOC*═*O), 158.2_3_ (NCN), 156.1_3_ (NHC═OO), 155.4_3_ (CNH_2_), 149.8_0_ (CONH), 139.1_9_ (NC4 ═C5N), 135.9_0_, 129.7_1_, 128.2_9_, 126.34 (aromatic carbons), 107.81 (NC4═C5N), 88.92 (NCH), 86.07 (CHCH_2_), 78.22 (C(CH_3_)_3_), 74.67 (CH_2_OH), 69.7_3_ (CHOH), 63.4_6_ (*C*H_2_OH), 56.2_8_ (*C*HNH), 37.5_2_ (*C*H_2_Ph), 36.0_2_ (N*C*H_3_) 28.6_2_ (C(*C*H_3_)_3_). Elemental analysis calcd for C_25_H_33_AuN_6_O_9_ (%): C 39.59, H 4.39, N 11.08; found (%): C 39.67, H 4.64, N 10.98. MALDI‐MS: (CH_3_OH) m/z: 668.62037 Da attributable to [C_21_H_23_AuN_6_O_7_] ^+^.

### 4.14 Biological Evaluation Assays

#### 4.14.1 MTT Assay

The in vitro anticancer activities of all the target compounds were determined by MTT assay (Merck Life Science S.r.l., Milano, Italy) [54]. Shortly, cells were seeded and grown in full medium, then starved in serum free medium (24 h). Next, cells were treated with the studied complexes (concentrations range: 10–200 µM), for 72 h. Then MTT (3‐(4,5‐dimethylthiazol‐2‐yl)−2,5‐diphenyl tetrazolium bromide, final concentration 0.5 mg/mL) was added and cells were incubated 2 h at 37 °C. At the end, cells were lysed using 200 μL of DMSO and the optical density measured (570 nm). Results are represented as percent (%) of basal absorbance and IC_50_ values ± standard deviations were calculated using GraphPad Prism 10 (GraphPad Software, La Jolla, CA, USA).

#### 4.14.2 TUNEL Assay

Nuclear DNA fragmentation was detected by the TUNEL assay, according to the guidelines of the manufacturer (One‐step TUNEL Assay Kit (Green, FITC), MBS2567761, MyBioSource, Inc., San Diego, CA 92 195−3308 USA). Cells were grown on glass coverslips (25.000 cells) and, after exposure to the studied complex at the concentration equal to the calculated IC_50_ value or DMSO (solvent), PBS washed and methanol‐fixed (−20 °C, 20 min). The TUNEL reaction mixture was then added and, after incubation at 37 °C, for 35 min, protected from light, cells were washed, incubated with DAPI (Sigma, 0.2 mg/mL, 5 min, dark) and then PBS washed again and observed under a fluorescence microscope (Leica DM6000; 20× magnification, λ_ex/em_ maxima of 490/515 nm for CF488A or 350/460 nm for DAPI). Representative fields of three independent experiments are shown.

#### 4.14.3 Mitochondrial Membrane Potential (MMP) Assessment

To determine the mitochondrial membrane potential (MMP or ΔΨ_M_), the fluorescent dye JC‐1 (5,5^′^,6,6^′^‐tetrachloro‐1,1^′^,3,3^′^‐tetraethylbenzimidazolylcarbocyanine iodide, Thermo Fisher Scientific S.p.A., Monza, Italy) was adopted. In cells with intact mitochondria, the high membrane potential allows a high accumulation of the dye within the mitochondria as aggregates that emit a red fluorescence (λ_ex/em_ = 644/665), whereas in cells with damaged mitochondria the increased membrane permeability results in a loss of the electrochemical potential. Under these conditions, the dye remains in the cytoplasm as green‐fluorescent monomers (λ_ex/em_ = 490/515 nm) and, at the same time, there is a significant decrease in red fluorescence (10.1016/j.bcp.2015.07.008). Thus, the evaluation of the red‐to‐green fluorescence ratio permits the estimation of the MMP and, ultimately, the impairment of the mitochondrial compartment. Briefly, cells were seeded in full medium and then treated for 24 h with **L2AuOAc** (17 μM) or only the solvent (DMSO). After medium removal, cells were incubated with a JC‐1 reagent solution (500 nM, final concentration) at 37 °C for 15 min. Next, cells where PBS washed and fixed with ice‐cold methanol (−20 °C, 15 min). After three washes with a PBS solution added with 0.01% Triton X‐100, cells were incubated with DAPI (4^′^,6‐diamidino‐2‐phenylindole, Merck Life Science S.r.l., Milano, Italy) dye for 10 min (room temperature). Finally, cells were washed with PBS and observed by the means of a Leica fluorescence microscope (Leica DM 6000). Images acquisition and processing were made using LAS‐X software (version 3.5.7.23225)

#### 4.14.4 Intracellular ROS Measurement

Intracellular ROS levels were measured by the means of dihydro‐2′,7′‐dichlorofluorescein diacetate (H_2_DCF‐DA) assay. MDA‐MB‐231 breast cancer cells were plated onto a black 96‐well plate, then treated with **L2AuOAc,** at the indicated concentrations, or vehicle (DMSO) for 24 h and incubated. Next, cells were PBS washed (37 °C) and incubated with 25 μM of a H_2_DCF‐DA solution (Merck Life Science S.r.l., Milano, Italy). After a final PBS wash, the fluorescence intensity was quantified using a microplate reader (λ_ex/em_ = 485/535 nm). The percentage of ROS production (% ROS) under the complex treatment was calculated versus the vehicle‐treated cells.

#### 4.14.5 Caspases Assay

Caspases 3/7, 8 and 9 activities were determined using the Caspase‐Glo 3/7, 8 and 9 Assay Systems (Promega Corporation, Madison, WI). Cells were grown in white‐walled 96‐well plate and treated with **L2AuOAc** at 17 μM. The Caspase‐Glo reagent and 96‐well plate containing cells were equilibrated to room temperature. Then, 100 µL of the different caspases’ reagent were added to each well (ratio 1:1) of the 96‐well plate, containing blank, negative control cells and treated cells. Plate was gently mixed using a shaker (300–500 rpm) for 2 min at room temperature. The luminescence was measured using a plate‐reading luminometer (Synergy H1 Hybrid Reader, BioTek) for 15 min to 3 h (λex = 490 nm, λem = 510–570 nm). Results were represented as signal‐to‐noise ratios. Background readings were acquired from wells containing culture medium without cells.

### 4.15 Virus Infection and Titration

#### 4.15.1 Cell Cultures and NHC‐Complexes Treatment

BEAS‐2B human bronchial epithelial cells (ATCC) were grown in DMEM medium, supplemented with 10% fetal bovine serum (FBS), 0.3 mg/ml glutamine, 100 U/ml penicillin and 100 μg/ml streptomycin. Au and Ag‐complexes were dissolved in DMSO and then diluted to the final concentration in cell‐culture medium. For the evaluation of antiviral activity, complexes were added, at the final concentration of 10 μM, after 1h‐infection and left for the 24 h of the infection. Oseltamivir carboxylate (Merck), 1 μM, was included as a control [55].

#### 4.15.2 Virus Infection

Influenza virus A/Puerto Rico/8/34 H1N1 (PR8) was grown in the allantoic cavities of 10 days old embryonated chicken eggs and harvested after 48 h. To perform infection, epithelial cells were challenged with PR8 at a multiplicity of infection (m.o.i.) of 0.1 for 1h at 37 °C, washed with PBS and incubated with medium with 2% FBS for 24 h.

#### 4.15.3 Quantitative Reverse Transcription‐PCR (qRT‐PCR)

Viral RNA (for HA gene) was determined in the supernatants of infected cells, with HA‐specific primers, by quantitative Reverse‐transcription PCR (qRT‐PCR); standard curves using plasmid standards allowed viral RNA to be quantified in Log_10_ copies/mL, as described previously [56].

#### 4.15.4 Western Blot Analysis

Cells were lysed with lysis buffer supplemented with phenylmethylsulfonyl fluoride (PMSF) and protease inhibitor mixture (Sigma–Aldrich) for 30 min on ice. Protein concentration was determined with DC Protein Assay (Bio‐Rad). Then equal amounts of protein were analysed by SDS‐PAGE followed by Western blot. The proteins were visualized using the following primary and secondary (horseradish peroxidase‐conjugated) antibodies: anti‐Influenza (Merck Millipore), anti‐GAPDH (Santa Cruz Biotechnology); anti‐goat, anti‐mouse (Bethyl Laboratories). Blots were developed with the WesternBright ECL HRP substrate (Advansta) and densitometric analysis was performed using ImageJ software.

#### 4.15.5 Statistical Analysis

Experiments were carried out at least three times, each performed in duplicate. Statistical analysis was performed using a two‐tailed Student’s test or one‐way ANOVA followed by Dunnett’s multiple comparison test. A *p* value < 0.05 was considered statistically significant. Standard deviations are shown

## Funding

This work was supported by PRIN 2022 PNRR, Code P20222BLAZ—Enhanced pharmacological activity of noble metal carbene‐N‐heterocyclic complexes by oligopeptide counterion (Grant CUP MASTER: D53D23016900001), PRIN 2022, Code 2022HARH5W—HyMTA (Hybrid Multi‐Target Agents) Synthesis and biological evaluation of chimeric hybrid molecules containing NHC‐metal complexes and carbazole moieties, as innovative multi‐target anticancer and antiviral agents (Grant CUP MASTER: C53D23004490001), Italian Ministry of Health‐Ricerca Corrente.

## Conflicts of Interest

The authors declare no conflicts of interest.

## Data Availability

The data that support the findings of this study are available from the corresponding author upon reasonable request.
